# Anthocyanin-Rich *Aronia melanocarpa* Supplementation Modulates Intestinal Barrier Biomarkers and Stress-Axis Response in a Randomized Controlled Trial

**DOI:** 10.3390/nu18213584

**Published:** 2026-10-29

**Authors:** Hanna Dziewiecka, Joanna Ostapiuk-Karolczuk, Anna Kasperska, Justyna Cichoń-Woźniak, Marta Pasek, Sabina Kaczmarczyk, Małgorzata Reysner, Piotr Basta, Anna Skarpańska-Stejnborn

**Affiliations:** 1 Department of Biological Sciences, Faculty of Sport Sciences in Gorzow Wielkopolski, Poznan University of Physical Education, Estkowskiego 13, 66-400 Gorzow Wielkopolski, Poland; j.ostapiuk@awf-gorzow.edu.pl (J.O.-K.); annakasperska.awf@gmail.com (A.K.); cichonjustyna95@gmail.com (J.C.-W.); m.pasek@awf-gorzow.edu.pl (M.P.); iskra@awf.poznan.pl (S.K.); maly197@interia.pl (P.B.); ankass@poczta.onet.pl (A.S.-S.); 2 Department of Clinical Anesthesiology and Pain Management, Poznan University of Medical Sciences, 61-701 Poznań, Poland; reysner.malgorzata@icloud.com

**Keywords:** *Aronia melanocarpa*, anthocyanins, intestinal permeability, gut barrier, cortisol, elite athletes

## Abstract

Background: Anthocyanin-rich berries may modulate intestinal barrier-related processes and physiological stress responses, but evidence from human studies remains limited. Methods: This randomized, double-blind, placebo-controlled trial included 15 elite male rowers (Aronia group, n = 7; placebo group, n = 8). Participants received *Aronia melanocarpa* supplementation or placebo for eight weeks and performed a standardized 2000-m rowing ergometer test before and after the intervention. Circulating claudin-3 (CLDN-3), lipopolysaccharide-binding protein (LBP), intestinal fatty acid-binding protein (I-FABP), and cortisol concentrations were assessed before exercise, immediately after exercise, and following 1 h of recovery. Results: A significant Group × Time interaction was observed for CLDN-3 (*p* = 0.0463). After eight weeks, pre-exercise CLDN-3 concentrations were lower in the Aronia group than in the placebo group (*p* = 0.0367). Lower pre-exercise LBP concentrations (*p* = 0.0125) and lower cortisol concentrations at 1 h post-exercise (*p* = 0.0079) were also observed in the Aronia group compared with placebo. No significant supplementation-specific effect was observed for I-FABP. Conclusions: Eight weeks of *Aronia melanocarpa* supplementation was associated with differences in selected intestinal barrier-related biomarkers and post-exercise cortisol concentrations in elite male rowers. However, these findings were primarily related to specific sampling time points and should not be interpreted as evidence of improved intestinal barrier function. Given the small sample size and potential dietary confounding, the results are preliminary and require confirmation in larger randomized controlled trials.

## 1. Introduction

Strenuous physical exercise can induce transient disturbances in gastrointestinal integrity and function, commonly described as exercise-induced gastrointestinal syndrome (EIGS) [1]. These disturbances result from interacting mechanisms, including intestinal hypoperfusion, oxidative stress, inflammatory signaling, and neuroendocrine activation [1]. Exercise-associated alterations can be investigated using complementary circulating biomarkers. Claudin-3 (CLDN-3) is a component of epithelial tight junctions [2], intestinal fatty acid-binding protein (I-FABP) reflects enterocyte injury [3], and lipopolysaccharide-binding protein (LBP) is involved in endotoxin-related inflammatory responses [2]. Although these biomarkers represent distinct physiological processes and do not directly measure intestinal permeability, their combined assessment may provide insight into gastrointestinal responses to exercise. High-intensity exercise also activates the hypothalamic–pituitary–adrenal axis, resulting in changes in cortisol concentrations that reflect neuroendocrine stress responses [1,4].

Dietary polyphenols, particularly anthocyanins, have attracted interest as potential modulators of intestinal barrier-associated processes through their antioxidant, anti-inflammatory, and microbiota-related activities [5]. However, evidence supporting their effectiveness in athletes remains limited. In their recent joint position statement, Costa et al. (2025) highlighted the insufficient evidence supporting anthocyanin supplementation for managing EIGS [6]. Although anthocyanin-rich blackcurrant extract attenuated exercise-associated increases in I-FABP, no significant effects were observed on functional intestinal permeability or endotoxin-related biomarkers, including LBP and soluble CD14 [7]. These findings suggest that anthocyanins may influence selected aspects of intestinal epithelial injury without necessarily improving overall barrier function. Moreover, the International Society of Sports Nutrition position stand (Gonzalez et al., 2026) emphasized that exercise-induced oxidative signaling contributes to physiological adaptation, indicating that attenuation of exercise-related responses should not automatically be considered beneficial [8].

*Aronia melanocarpa* (black chokeberry) is a rich source of anthocyanins, predominantly cyanidin glycosides, together with proanthocyanidins, flavonols, and phenolic acids. Previous investigations have demonstrated its potential antioxidant and anti-inflammatory activities in both clinical and athletic populations [5]. Skarpańska-Stejnborn et al. (2014) reported reduced TNF-α concentrations following eight weeks of chokeberry juice supplementation in elite rowers [9], while Stankiewicz et al. (2023) investigated inflammatory responses following Aronia supplementation in young football players [10]. Our previously published systematic review (Kaczmarczyk et al.) included 57 studies, of which 45 involved animal models and 12 involved humans [5]. Importantly, only three human studies evaluated intestinal parameters, primarily focusing on gut microbiota composition [11,12,13]. Although experimental studies suggest that Aronia may influence tight junction-associated mechanisms, evidence concerning its effects on intestinal barrier-related biomarkers in humans remains insufficient [14,15].

Consequently, several important research gaps remain. First, the effects of prolonged Aronia supplementation on circulating markers reflecting different aspects of intestinal physiology have not been adequately investigated in humans. Second, it remains unclear whether supplementation modifies resting biomarker concentrations or their responses to acute high-intensity exercise. Third, the potential influence of Aronia supplementation on exercise-associated cortisol responses has rarely been evaluated alongside gastrointestinal biomarkers. Addressing these gaps may help clarify whether the biological activities attributed to Aronia-derived polyphenols are reflected in measurable physiological responses in highly trained athletes.

Therefore, this randomized, double-blind, placebo-controlled trial aimed to explore the effects of eight weeks of anthocyanin-rich *Aronia melanocarpa* supplementation on circulating CLDN-3, I-FABP, LBP, and cortisol concentrations in elite male rowers performing a standardized 2000-m rowing ergometer test. We hypothesized that supplementation would modulate selected intestinal barrier-related biomarkers and attenuate cortisol responses to strenuous exercise. Given the limited human evidence, the study was intended to provide preliminary, hypothesis-generating findings rather than definitive evidence of improved intestinal barrier function.

The scientific contribution of the present study lies in its exploratory evaluation of exercise-related intestinal barrier biomarkers and cortisol responses following Aronia supplementation in elite athletes, a context in which controlled human evidence remains limited. Rather than providing definitive evidence of efficacy, the findings may help identify candidate outcomes and methodological considerations for future adequately powered trials.

## 2. Materials and Methods

This study represents a physiological model of stress response in elite athletes, using an established 2000 m rowing protocol to elicit maximal HPA and gastrointestinal activation [16,17]. In accordance with the CONSORT guidelines, the trial methodology and reporting were structured to ensure transparency, completeness, and methodological rigor [18].

### 2.1. Study Design

**Figure 1:**
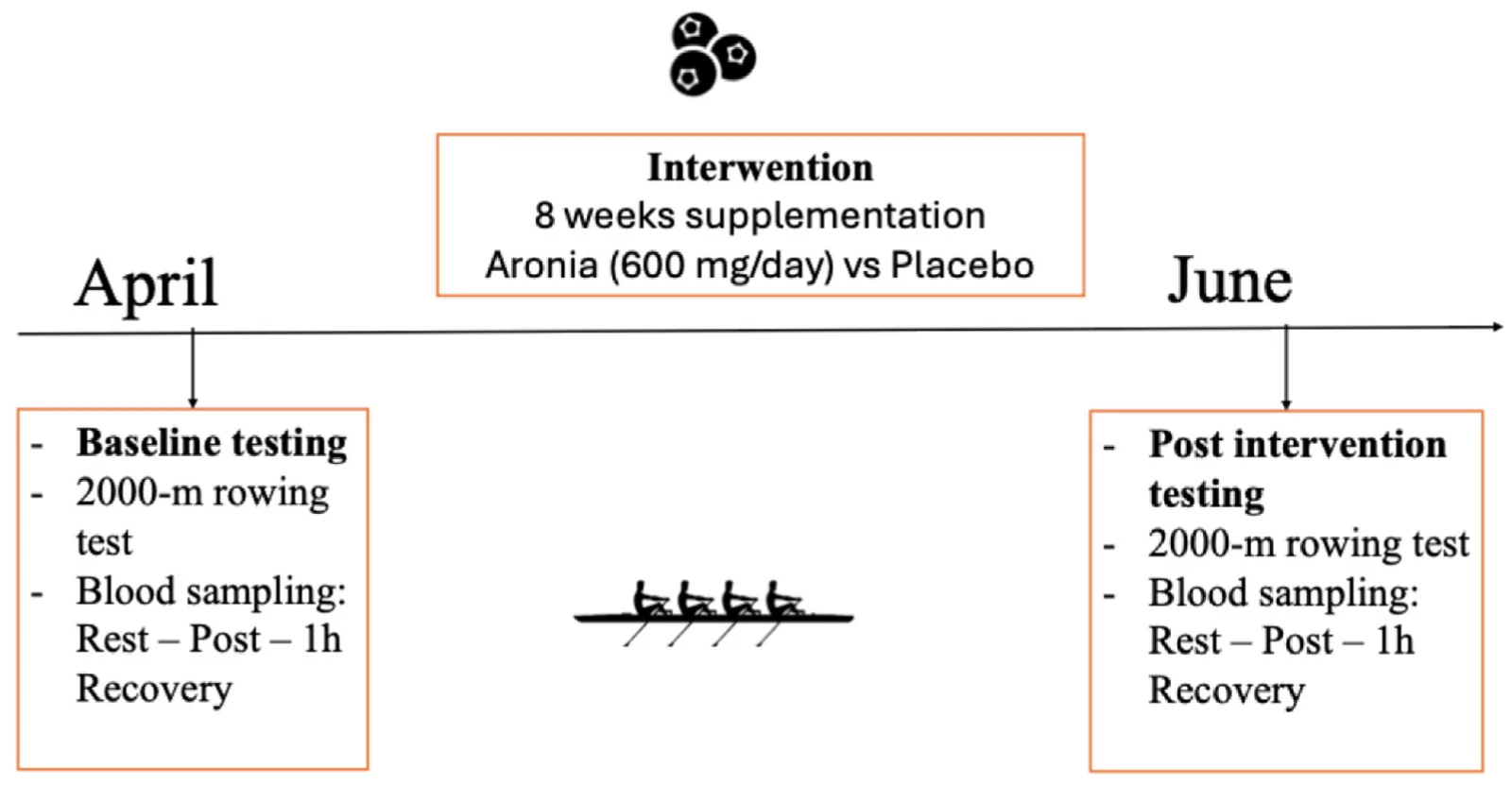
Study design and experimental timeline of the randomized, double-blind, placebo-controlled trial.

This prospective, double-blind, randomized controlled trial (RCT—Figure 1) was conducted at a single center in Poland. The study protocol received approval from the Bioethics Committee of the Poznan University of Medical Sciences on 23 June 2022 (protocol number 390/22). The trial was retrospectively registered on 14 November 2023 at ClinicalTrials.gov (NCT06133751). Written informed consent was obtained from all participants before their enrollment. The study was conducted in accordance with the Declaration of Helsinki. A parallel-group design was applied. Although a cross-over design can improve statistical power and reduce inter-individual variability, this approach was not feasible in the present study due to the characteristics of the study population. The trial was conducted during the preparation phase of elite rowers, a period during which training load, competition schedule, and recovery phases varied substantially over time. Implementing a cross-over design would have required an extended washout period, during which athletes would have been exposed to different training intensities and seasonal workloads. These variations could have introduced substantial confounding effects, making it impossible to attribute observed changes exclusively to the intervention. Therefore, a parallel-group randomized controlled design was selected as the most appropriate approach within the constraints of the athletes’ annual training and competition calendar.

### 2.2. Participants

Male members of the Polish National Rowing Team were considered for enrolment. Fifteen elite rowers (male) from the Polish National Rowing Team, actively competing at national and international level, voluntarily participated in this randomized, double-blind, placebo-controlled trial. Eligibility criteria included: at least five years of systematic rowing-specific training at national or international competitive level, including regular participation in endurance, technical, and strength training session, a minimum weekly training volume of 240 min, current membership in the Youth National Rowing Team of Poland, and completion of a 2000-m rowing ergometer test on two designated test dates. Exclusion criteria: history of gastrointestinal disease, chronic inflammatory or metabolic disorders, acute infection within the previous four weeks, current musculoskeletal injury limiting training participation, use of antibiotics, probiotics, anti-inflammatory medication, or nutritional supplements known to affect gastrointestinal function within four weeks prior to enrollment.

### 2.3. Supplementation Protocol

The 8-week duration was selected to provide adequate exposure to anthocyanin-rich supplementation and to be compatible with the athletes’ training macrocycle. The 600 mg/day dose (18% anthocyanins) reflects a standardized extract amount within ranges used in previous human studies and was feasible for daily use in elite athletes [9,10]. The dose was split into three capsules taken with meals to improve tolerability and adherence. Participants consumed three capsules daily (one with each meal) for eight weeks. The intervention consisted of concentrated black chokeberry extract (standardized to 18% anthocyanins), while the control group received placebo capsules. Each chokeberry capsule contained 200 mg of dry black chokeberry extract in addition to chokeberry fiber, magnesium salts of stearic acid (E460b), silicon dioxide (E551), hydroxypropyl methylcellulose (capsule shell), and titanium dioxide (E171, shell color). To maintain blinding, both active and placebo capsules were manufactured to be indistinguishable in size, color, taste, and external appearance. The characteristic dark coloration of *Aronia* extract was masked by using opaque capsules, and both supplements were dispensed in identical coded containers. Both the supplement and placebo were manufactured and blinded by MBL BIOTRADE (Poznań, Poland).

### 2.4. Randomization and Concealment

Participants were enrolled by a member of the research team and randomly allocated to the Aronia (n = 7) or placebo (n = 8) group using a computer-generated simple randomization sequence (randomizer.org). The sequence was generated by an independent researcher who was not involved in data collection or statistical analysis. No stratification by rowing position or training load was applied. The supplement manufacturer (MBL BIOTRADE, Poznań, Poland) prepared identical, sealed, and numbered tubes containing either *Aronia melanocarpa* or placebo capsules. The numbered tubes were assigned to participants by an independent investigator in accordance with the previously generated randomization sequence. Group allocation codes were held by an independent researcher and remained concealed from participants and the research team until completion of data collection and statistical analyses. Participants were instructed to take the capsules with water. Supplementation adherence was assessed by counting the remaining capsules at the end of the eight-week intervention and exceeded 95% in both groups.

### 2.5. Procedures

The athletes underwent the first exercise test in April 2023 during a residential national-team training camp conducted as part of the direct pre-competition preparation phase for the international rowing season. The camp followed a standardized national-team training program consisting of supervised endurance, technical, speed, and strength sessions performed under identical coaching, nutritional, and recovery conditions. After eight weeks of chokeberry or placebo supplementation, the second testing session was conducted in June during the same training cycle. Each participant completed a standardized 2000-m rowing ergometer test (Concept II, Morrisville, VT, USA) at both time points. Training sessions were prospectively recorded daily, including intensity, volume, and type (endurance, technical, speed, strength, or jogging). Training intensity was classified according to blood lactate thresholds routinely used in the national team training system. Sessions performed below 4 mmol/L were categorized as low-intensity endurance training, whereas sessions ≥ 4 mmol/L were classified as high-intensity endurance training (Table 1). As the study involved members of the national rowing team, the training process was not subject to experimental modification to avoid any disruption of the preparation program. Both the control and supplemented groups followed an identical training schedule, and compliance with the prescribed training loads was strictly maintained and verified throughout the camp. The seven-day training program preceding each 2000-m test is shown in Table 1. Throughout the intervention period, all athletes resided at the same training camp, followed the same coaching schedule, and were exposed to identical environmental and recovery conditions, thereby minimizing non-exercise-related variability between participants.

The 2000 m rowing ergometer test was used as a validated model of acute exercise-induced stress, eliciting maximal cardiovascular, endocrine, and gastrointestinal responses in elite athletes. In the first trial, the supplemented group achieved a mean time of 373 ± 8 s and a mean power output of 432 ± 30 W, while the placebo group recorded 377 ± 7 s and 418 ± 24 W.

In the second trial, following the supplementation period, the results were 373 ± 7 s and 430 ± 25 W for the supplemented group, and 374 ± 6 s and 423 ± 27 W for the placebo group (no statistical differences occurred within between groups). Before each test, participants performed a standardized five-minute warm-up. Athletes were highly motivated to perform the test at maximum effort, as the results influenced their selection for the championship team. All participants followed the same structured training program, irrespective of their allocation to the Aronia or placebo group. Training schedules were documented at the group level, and individual training-load measurements were not collected.

### 2.6. Outcome Measure

Blood samples were collected from the antecubital vein during both testing sessions, before exercise after an overnight fast (10–12 h), immediately after exercise, and after 1 h of recovery. After the fasting blood collection, participants consumed their habitual pre-exercise breakfast. Following collection and clot formation, blood samples were centrifuged, and serum was separated and stored at −80 °C until analysis. Samples underwent a single freeze–thaw cycle for the assays.

Serum I-FABP, LBP, and CLDN-3 concentrations were measured using commercial ELISA kits (SunRed Biotechnology Company, Shanghai, China; catalog numbers 201-12-1542, 201-12-0361, and 201-12-2303, respectively). The respective assay ranges were 0.3–80 ng/mL, 0.2–60 µg/mL, and 0.5–60 ng/mL, with analytical sensitivities of 0.156 ng/mL, 0.135 µg/mL, and 0.302 ng/mL. Manufacturer-reported intra- and inter-assay CVs for LBP were <10% and <12%, respectively. Cortisol was measured using a DRG ELISA kit (DRG Instruments GmbH, Marburg, Germany; catalog number EIA-1887; assay range: 1.3–800 ng/mL; analytical sensitivity: 1.3 ng/mL; intra-assay CV: 3.2–8.1%). The manufacturers did not provide intra- or inter-assay CVs for I-FABP and CLDN-3, an inter-assay CV for cortisol, or LLOQs for I-FABP, LBP, and CLDN-3 in the supplied instructions. Kit lot numbers were not available in the study records. All measurements were within the respective assay ranges, with no values below or above these ranges; therefore, no exclusions or substitutions were required. Absorbance was read using a SPECTROstar Nano spectrophotometer (BMG LABTECH, Offenburg, Germany).

Given the circadian rhythm of cortisol secretion, blood sampling was performed at standardized time points (07:00 and approximately 11:00–12:00) during both testing sessions to minimize the influence of diurnal variation.

### 2.7. Anthropometric Measurements

The study also included anthropometric measurements, including assessment of body composition and weight using bioelectrical impedance (Tanita BC 418, Tokyo, Japan), with an accuracy of 0.05 kg. Analysis was performed in the morning, after an overnight fast, during both the first and second stages of the study. Height was measured using a stadiometer (SECA 213, Hamburg, Germany). Participants wore only underwear during the measurements.

### 2.8. Dietary Intake

Dietary intake was recorded on the day preceding each test and on each test day. Dietary intake was assessed at two time points: during the 24 h preceding the exercise tests (to capture overall nutritional background) and on the test day, when only the pre-exercise breakfast was recorded. Breakfast was specifically chosen because it represents the last meal before testing and thus the most direct nutritional factor influencing acute exercise and hormonal responses. Participants documented their food intake in dietary diaries, which a dietitian supervised. Nutritional data, including total energy, protein, fat, carbohydrate, and fiber intake, were analyzed using DietetykPro software 2.0.13 (DietetykPro, Wrocław, Poland). Meals were prepared in a centralized canteen, allowing the dietitian to verify recipes and ensure accurate dietary monitoring. In addition, participants were asked to avoid substances known to influence exercise-induced immune responses (i.e., glutamine, nonsteroid anti-inflammatory drugs, bovine colostrum, curcumin, and probiotics) while involved in the study.

### 2.9. Statistical Analysis

Statistical analyses were performed using GraphPad Prism 9 (GraphPad Software, Boston, MA, USA). Data are presented as mean ± standard deviation (SD). Normality of data distribution was assessed using the Shapiro–Wilk test.

Biomarker responses to the exercise test were analyzed using two-way repeated-measures ANOVA to assess the effects of Group (Aronia supplementation vs. placebo), Time (pre-exercise, immediately post-exercise, and 1 h post-exercise), and the Group × Time interaction. Analyses were performed separately for the testing sessions conducted before and after the eight-week supplementation period. When appropriate, the Greenhouse–Geisser correction was applied. Significant ANOVA effects were followed by Tukey’s multiple-comparisons post hoc tests to identify differences between individual time points and between groups. Additional selected within-group comparisons between pre- and post-supplementation measurements were performed to evaluate changes over the eight-week intervention period. Dietary intake variables (energy, protein, fat, carbohydrate, and fiber) were compared between testing sessions using paired *t*-tests and between groups using unpaired *t*-tests. Effect sizes were expressed as Cohen’s d and interpreted as small (0.2), moderate (0.5), or large (0.8), according to Cohen’s criteria [19]. Exploratory multiple linear regression analyses were performed to investigate potential associations between dietary intake and selected gastrointestinal and neuroendocrine biomarkers, including CLDN-3, LBP, and cortisol. These analyses were considered hypothesis-generating and were not intended to establish causal relationships or exclude dietary confounding. Given the small sample size (n = 15), additional multivariable adjustment of the primary biomarker models for dietary covariates was not performed because of the risk of unstable parameter estimates and overfitting. Therefore, the potential contribution of dietary factors to the observed biomarker responses cannot be excluded.

#### Statistical Considerations

No formal a priori sample-size calculation was performed because recruitment was constrained by the availability of eligible elite national-team rowers during the designated training-camp period. Therefore, all eligible and available athletes were invited to participate. In accordance with methodological recommendations by Lakens (2022) [20], and due to the lack of consistent effect-size estimates for acute gastrointestinal, hormonal, and neuroendocrine responses in elite athletes, a sensitivity analysis was performed instead of a conventional a priori power analysis.

A sensitivity analysis conducted using G*Power 3.1 (Faul et al., 2007) [21] indicated that, for a repeated-measures within–between interaction (two groups, three repeated measurements, α = 0.05, power = 0.80), the final sample size (N = 15) corresponded to a minimum detectable effect size of Cohen’s f = 0.45 under the specified assumptions. Thus, the study was primarily sensitive to relatively large interaction effects, whereas smaller effects may have remained undetected.

## 3. Results

### 3.1. Participant Flow and Characteristics

**Figure 2:**
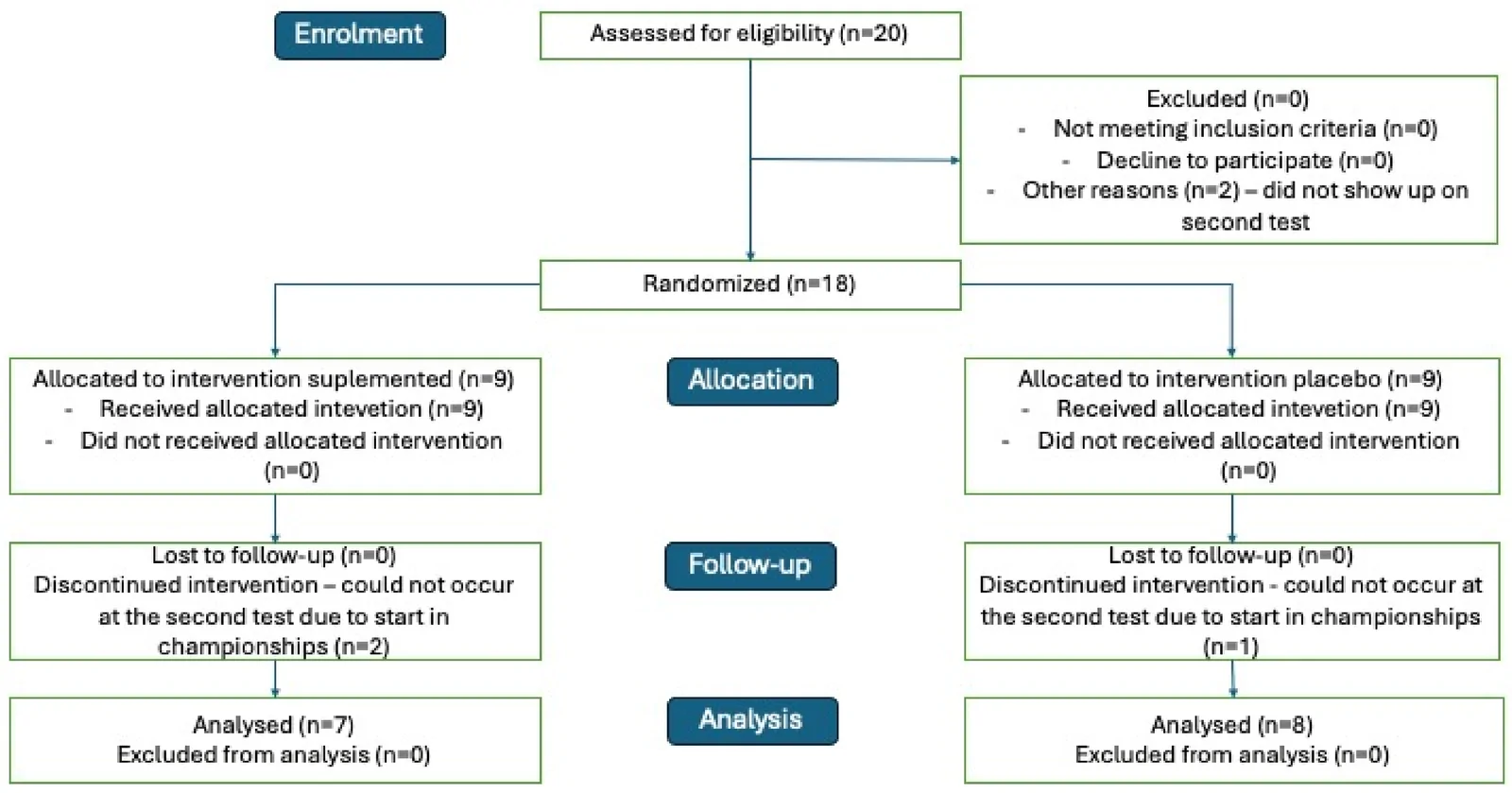
Flow chart diagram of participants in the study.

Two of the twenty athletes assessed for eligibility did not appear on the second test. The remaining eighteen were randomly allocated to two groups. Three participants were lost to discontinued intervention; they did not appear on the second test after supplementation, as they had competed in the championships. The remaining fifteen participants were analyzed, comprising 7 from the supplemented group and 8 from the placebo group, as shown in Figure 2.

### 3.2. Anthropometric Measurements

No statistically significant differences were observed between groups for any of the measured parameters, as detailed in Table 2.

### 3.3. Dietary Intake

The analysis of dietary intake revealed several statistically significant between-group differences, as presented in Table 3. During the 24 h preceding the exercise tests, protein intake was significantly higher in the Aronia group than in the placebo group both before supplementation (222.39 ± 43.67 vs. 171.03 ± 34.51 g, *p* = 0.03) and after supplementation (199.31 ± 41.95 vs. 167.23 ± 24.11 g, *p* = 0.03).

Following the eight-week intervention, fat intake was significantly higher in the Aronia group than in the placebo group (122.37 ± 37.37 vs. 86.82 ± 12.99 g, *p* = 0.01), whereas fiber intake was significantly lower (29.19 ± 13.64 vs. 48.09 ± 23.24 g, *p* = 0.048). Within-group analysis showed a decrease in fat intake in the placebo group (*p* = 0.036), whereas protein and fiber intake did not change significantly in either group. Energy and carbohydrate intake did not differ significantly between groups or testing sessions.

Regarding dietary intake on the exercise test days, no statistically significant between-group differences or within-group changes were observed for energy, protein, fat, carbohydrate, or fiber intake before exercise.

Exploratory multiple linear regression analysis identified a negative association between protein intake and CLDN-3 concentrations (β = −0.0415, *p* = 0.033). CLDN-3 was not significantly associated with fat or fiber intake. No statistically significant associations were found between the dietary variables examined and LBP or cortisol concentrations.

Dietary fiber intake was calculated from food records and did not include fiber derived from the placebo capsules, as its exact quantity was unavailable.

### 3.4. Results of Biomarker Response to Exercise Testing

The results of the two-way ANOVA for CLDN-3, LBP, I-FABP, and cortisol are summarized in Table 4.

#### 3.4.1. Claudin-3 (CLDN-3)

**Figure 3:**
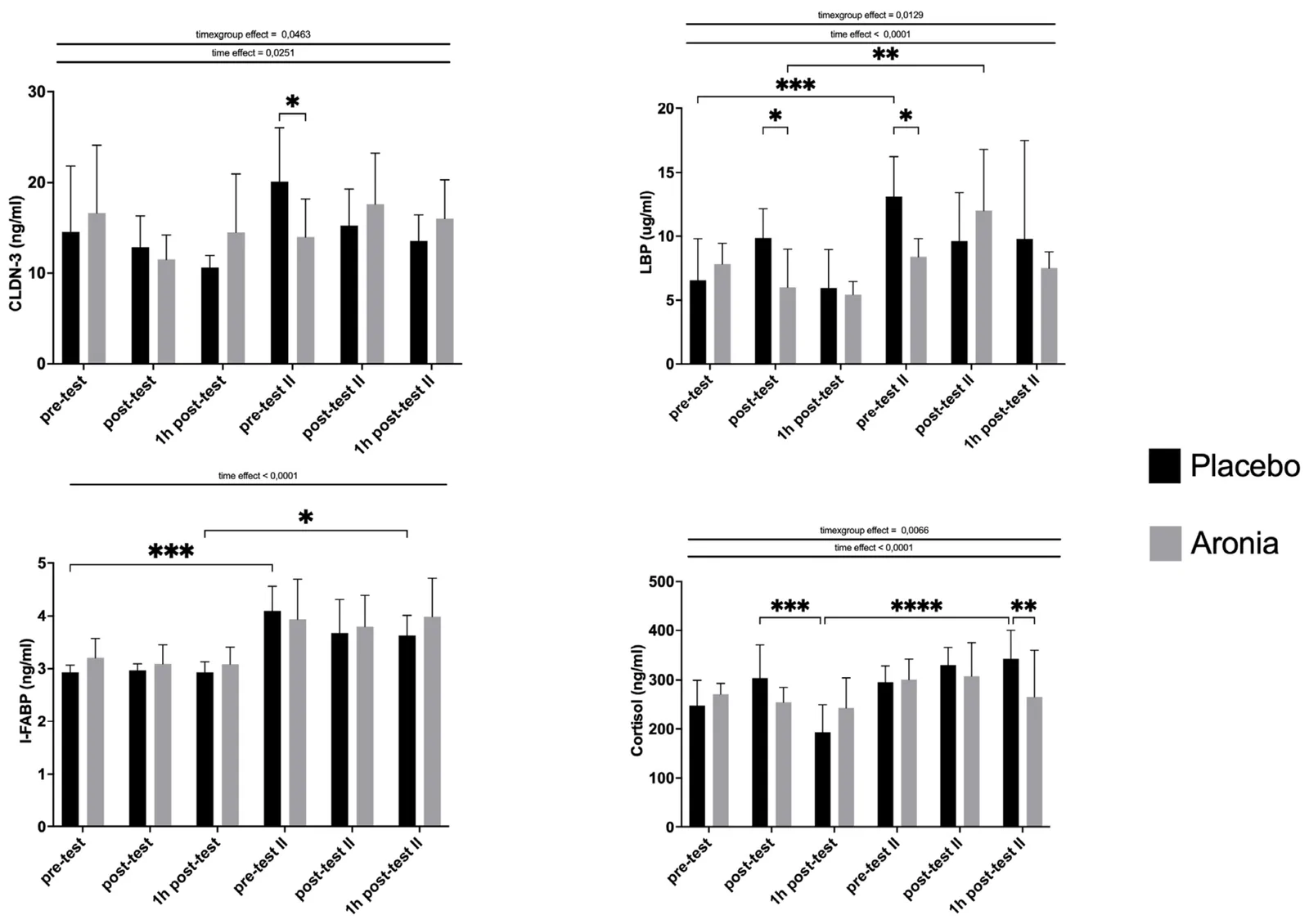
Effects of the 2000-m rowing test on CLDN-3, LBP, cortisol, and I-FABP measured at Rest, Post-exercise, and 1 h Recovery during Test I (pre-supplementation) and Test II (post-supplementation). Footnotes: Values are presented as mean ± SD, significant differences: * *p* < 0.05; ** *p* < 0.01; *** *p* < 0.001; **** *p* < 0.0001.

Post hoc analysis revealed no significant between-group differences before supplementation. Following the intervention, resting CLDN-3 concentrations were significantly higher in the placebo group than in the Aronia group (*p* = 0.0367, Cohen’s d = 1.30). No other significant pairwise differences were observed (Figure 3).

#### 3.4.2. Lipopolysaccharide-Binding Protein (LBP)

Before supplementation, baseline LBP concentrations were comparable between groups. Immediately after exercise, LBP concentrations increased in both groups, with higher values in the placebo group (*p* = 0.0401, Cohen’s d = 1.57), followed by a decline during recovery.

Following supplementation, resting LBP concentrations were higher in the placebo group than in the Aronia group (*p* = 0.0125, Cohen’s d = 2.24). Resting LBP also increased significantly in the placebo group compared with pre-supplementation values (*p* = 0.0090, Cohen’s d = 2.21). In the Aronia group, post-exercise LBP concentrations showed a nonsignificant increase compared with the pre-supplementation period (*p* = 0.0670, Cohen’s d = 1.66) (Figure 2).

#### 3.4.3. Intestinal Fatty Acid-Binding Protein (I-FABP)

No significant changes in I-FABP concentrations were observed before supplementation. Following the intervention, resting I-FABP concentrations in the placebo group were significantly higher than before supplementation (*p* = 0.0006, Cohen’s d = 4.25). Similarly, I-FABP concentrations at 1 h post-exercise were higher than the corresponding pre-supplementation values (*p* = 0.0227, Cohen’s d = 2.56) (Figure 3).

#### 3.4.4. Cortisol

Before supplementation, cortisol concentrations were comparable between groups, with a significant decrease during recovery in the placebo group (*p* = 0.0030, Cohen’s d = 1.92). Following supplementation, cortisol concentrations at 1 h post-exercise were significantly lower in the Aronia group than in the placebo group (*p* = 0.0079, Cohen’s d = 1.09). Recovery cortisol concentrations in the placebo group were also higher after supplementation than before supplementation (*p* < 0.001, Cohen’s d = 2.79).

## 4. Discussion

Controlled acute physiological stress induced by exercise was used in this study as a human model to investigate interactions between anthocyanin-rich supplementation and stress-responsive biological pathways. This study aimed to evaluate the effects of *Aronia* supplementation on gastrointestinal integrity and stress-related physiological pathways under controlled stress conditions. The present findings suggest that anthocyanin-rich black chokeberry supplementation may modulate exercise-induced alterations in gut barrier-related biomarkers and neuroendocrine responses. Although previous studies have reported transient increases in markers of intestinal permeability following a 2000-m rowing test [16], the present study did not demonstrate consistent time-dependent increases across all gut barrier biomarkers. This may reflect adaptive responses in highly trained athletes, in whom repeated exposure to high-intensity exercise can attenuate gastrointestinal perturbations.

Intestinal dysbiosis and increased permeability have been reported in several pathological conditions and are particularly relevant in athletes exposed to high training loads and limited recovery time. These alterations can contribute to gastrointestinal symptoms and impaired health status. Present study examined whether *Aronia* supplementation could modulate neuroendocrine and circulatory pathways associated with gastrointestinal dysfunction, focusing on biomarkers of stress, epithelial injury, and systemic inflammation.

The study revealed significant group- and time-related effects for CLDN-3 and LBP, with post hoc analyses indicating that the observed differences were primarily associated with pre-exercise biomarker concentrations after supplementation. In particular, the significant Group × Time interaction for CLDN-3 (*p* = 0.0463) suggests that its temporal response differed between the study groups. However, the significant between-group difference was observed specifically in pre-exercise CLDN-3 concentrations after eight weeks of supplementation (*p* = 0.0367), with lower values in the Aronia group than in the placebo group. These findings may indicate an association between chokeberry supplementation and resting intestinal barrier-related biomarker concentrations rather than a consistent protective effect across all exercise-related time points. Importantly, differences in dietary protein intake between groups may have contributed to the observed CLDN-3 responses and cannot be excluded as a potential confounding factor.

Consistent with Young et al. [22], circulating biomarkers related to exercise-induced gastrointestinal syndrome exhibit considerable biological variability, and their reliability and minimal detectable change may vary substantially, particularly for exercise-induced responses. Therefore, in the present study, CLDN-3, LBP, I-FABP, and cortisol were interpreted collectively as a biomarker cluster reflecting intestinal barrier and stress-axis modulation, rather than as isolated diagnostic indicators of clinically meaningful gastrointestinal injury [22]. We acknowledge that differences in training load between testing sessions may have influenced baseline biomarker concentrations. Moreover, a between-group difference in post-exercise LBP was already observed during the first testing session, prior to supplementation, indicating that randomization did not fully equalize this parameter at that time point. Consequently, the post-supplementation differences may partly reflect pre-existing variability between groups rather than solely the effect of the intervention. Despite these considerations, there remains a paucity of human studies directly examining the effects of chokeberry on intestinal permeability. Current evidence is largely derived from animal and in vitro models. Valdez et al. [14] demonstrated in vitro that *Aronia* enhances tight junction protein expression and improves epithelial barrier integrity, providing a rationale for human studies [14]. Animal research further supports these effects: *Aronia* supplementation improved gut barrier function and reduced colonic inflammation via modulation of the TLR4/NF-κB pathway in obese rats [23], and increased jejunal tight junction gene expression in pigs [15]. Although these findings are promising, the mechanisms through which *Aronia* influences intestinal barrier integrity remain incompletely understood. Due to its high anthocyanin content, *Aronia* supplementation has also been shown to modulate gut microbiota composition. Preclinical studies reported an increased abundance of beneficial microbial populations, particularly Bacteroides and Bacteroidetes, suggesting a potential role in promoting gastrointestinal health [24].

Mean protein intake in the Aronia group decreased over the supplementation period, although this change was not statistically significant. Exploratory regression analysis identified a negative association between protein intake and CLDN-3 concentrations (β = −0.0415, *p* = 0.033). Although this association does not establish causality, it suggests that dietary protein intake may have contributed to the observed variation in CLDN-3 concentrations. Consequently, the potential influence of dietary factors cannot be excluded, and the observed differences should not be attributed exclusively to chokeberry supplementation.

The different responses of I-FABP and CLDN-3 may reflect the distinct biological processes represented by these biomarkers. I-FABP is primarily associated with enterocyte injury, particularly in response to exercise-induced intestinal hypoperfusion and subsequent reperfusion, whereas CLDN-3 is a structural component of epithelial tight junctions involved in regulating intestinal barrier integrity. Anthocyanins may influence tight junction regulation through their antioxidant and anti-inflammatory properties without necessarily preventing acute enterocyte injury caused by strenuous exercise. Therefore, changes in CLDN-3 concentrations may occur independently of changes in I-FABP. However, the present study did not directly assess tight junction expression or intestinal permeability, and this mechanistic interpretation remains hypothetical.

To date, the effects of *Aronia* supplementation on gastrointestinal health in athletes have not been thoroughly examined. Evidence from studies on other polyphenol-rich interventions suggests potential benefits. For example, Nocella et al. [25] reported that a 30-day supplementation with 40 g of dark chocolate in elite football players resulted in significant reductions in circulating lipopolysaccharide (LPS), zonulin, and occludin compared with a control group. These findings indicate that polyphenol-rich supplementation may beneficially modulate markers of gut permeability and systemic inflammation in athletic populations [25]. Moreover, Valder et al. [26] reported a positive influence of diluted cloudy apple juice on CD14 in ultra-marathon runners. This finding is consistent with our observations, suggesting that polyphenol-rich interventions may modulate innate immune responses through pattern recognition receptors [26]. Conversely, cocoa did not affect I-FABP and LPS in endurance athletes [27]. The outcomes highlight the importance of polyphenols and their potential physiological effects. The present findings show that *Aronia* supplementation may influence pathways involved in gastrointestinal distress, potentially through modulation of gut microbiota composition. Gastrointestinal markers also showed time-dependent changes across the measured parameters, predominantly in the placebo group. I-FABP concentrations at baseline (pre-test) and recovery (1 h post-test) were significantly higher in the post-supplementation session compared with the pre-supplementation session. Similarly, baseline (pre-test) LBP levels were elevated in the post-supplementation session relative to the pre-supplementation session. These results differ from our previous findings, which showed that markers of endotoxemia and intestinal permeability decrease over the course of a training period [28]. Chantler et al. (2022) [29] proposed that the gut adapts to physical training, resulting in improved barrier function; however, our results do not align with this hypothesis.

In the supplemented group, cortisol levels were lower after 1 h of restitution compared to placebo. Cortisol, the final effector hormone of the HPA axis, plays a pivotal role in regulating metabolic, cardiovascular, and central nervous system responses to stress. The observed reduction in cortisol levels suggests that *Aronia* supplementation may modulate the HPA axis, thereby mitigating stress-related physiological responses [30]. Limited research exists on the impact of anthocyanins on stress hormone regulation. However, study examining the effects of a 6-week intake of anthocyanin-rich mulberry milk showed a positive influence on cortisol levels in working-age adults. These results show a potential role of anthocyanins in affecting endocrine stress responses [31]. Single doses of anthocyanins also lowered cortisol levels in school-aged children [32]. Nonetheless, the impact of exercise studies remains unclear. Intake of blueberries (another source of anthocyanins) did not alter cortisol levels after running [33]. The lower cortisol concentrations observed at 1 h post-exercise in the Aronia group suggest that supplementation may modulate the hormonal response during recovery. This finding provides a promising basis for further research into whether such changes translate into improved post-exercise recovery.

This randomized controlled trial provides novel human evidence that anthocyanin-rich *Aronia* supplementation modulates biomarkers of intestinal barrier integrity and stress-axis regulation under controlled physiological stress conditions. These findings extend current understanding of how dietary polyphenols may influence systemic adaptation to stress beyond their established antioxidant properties. Further research is warranted to elucidate mechanistic pathways and to determine the broader applicability of these effects in other populations.

### 4.1. Study Limitations

Several important limitations substantially restrict the interpretation of the present findings. The most critical limitation is the very small sample size (n = 15; Aronia: n = 7; placebo: n = 8), which considerably reduces statistical power and the precision of effect estimates. Consequently, the study is particularly vulnerable to both false-negative findings and unstable estimates of treatment effects. Moreover, statistically significant differences and large Cohen’s d values should be interpreted with considerable caution, as individual observations may disproportionately influence the results in such small groups. The limited sample size also increases the likelihood of baseline imbalances and restricts the ability of randomization to achieve comparability between groups.

Another important limitation is the potential influence of differences in training load between the pre- and post-supplementation testing periods. Variations in training volume and intensity may have affected inflammatory, gastrointestinal, and hormonal responses independently of supplementation, making it difficult to distinguish intervention-related effects from training-related adaptations. Furthermore, the assessment of multiple biomarkers across several time points and the use of numerous post hoc comparisons increase the risk of type I errors, particularly in a small exploratory study. Therefore, isolated statistically significant findings should not be interpreted as definitive evidence of supplementation efficacy. Pre-exercise meals were not standardized, and the biomarker analyses were not adjusted for dietary intake; therefore, dietary confounding cannot be excluded.

The interpretation of gastrointestinal outcomes is additionally limited by the indirect nature of the selected biomarkers. Although CLDN-3, I-FABP, and LBP provide complementary information on intestinal epithelial integrity, enterocyte injury, and endotoxin-related responses, they do not directly quantify intestinal permeability. The absence of a functional permeability test or a validated assessment of gastrointestinal symptoms prevents conclusions regarding clinically meaningful improvements in intestinal barrier function. In addition, the inclusion of only elite male rowers limits the generalizability of the findings to female athletes, other sporting disciplines, and less-trained populations. Taken together, these limitations indicate that the present results should be considered preliminary and hypothesis-generating. Although some biomarker differences were observed following Aronia supplementation, the current study cannot establish a definitive protective effect on intestinal barrier function or cortisol responses. Larger, adequately powered randomized controlled trials with standardized training loads, functional intestinal permeability assessments, and prespecified primary outcomes are required to confirm or refute these findings.

### 4.2. Future Directions

Despite its limitations, the present study provides preliminary data that may inform the design of future investigations into the effects of *Aronia melanocarpa* supplementation on exercise-induced gastrointestinal responses. The observed differences in CLDN-3, LBP, and cortisol suggest that these biomarkers may be useful candidates for further investigation, although their responsiveness to supplementation requires confirmation. Future randomized controlled trials should include larger samples, prospectively defined primary outcomes, and sample size calculations informed by the variability and effect estimates obtained in the present study. Particular attention should be given to controlling training load, dietary intake, and other potential confounding factors. Furthermore, combining circulating biomarkers with functional intestinal permeability tests, gastrointestinal symptom questionnaires, and direct measurements of endotoxin-related responses would provide a more comprehensive assessment of intestinal function. Future studies could also investigate different supplementation doses and durations, include female athletes, and evaluate whether biomarker changes are associated with gastrointestinal symptoms, recovery, or exercise performance. Such investigations would help determine the physiological and potential practical relevance of Aronia supplementation in athletic populations.

## 5. Conclusions

The present randomized, double-blind, placebo-controlled trial provides preliminary evidence that eight weeks of *Aronia melanocarpa* supplementation may influence selected intestinal barrier-related biomarkers and cortisol responses in elite male rowers exposed to high-intensity exercise. Following supplementation, resting CLDN-3 and LBP concentrations were significantly lower in the Aronia group than in the placebo group, suggesting potential modulation of tight junction-associated and endotoxin-related processes. In contrast, I-FABP showed a significant time effect without a significant Group × Time interaction, indicating that the observed changes cannot be attributed specifically to supplementation. Additionally, lower cortisol concentrations during post-exercise recovery in the supplemented group suggest a possible influence on the neuroendocrine stress response. However, these findings should be interpreted cautiously given the very small sample size, potential differences in training load, multiple statistical comparisons, and the indirect nature of the selected gastrointestinal biomarkers. Importantly, the results do not establish improved intestinal permeability or clinically meaningful gastrointestinal protection. Nevertheless, they provide a rationale for larger, adequately powered randomized controlled trials incorporating functional permeability assessments, gastrointestinal symptoms, and standardized training conditions to determine whether Aronia supplementation produces reproducible physiological benefits in athletes.

## Figures and Tables

**Table 1:** Training program 7 days before the tests.

**Test I**							
Days before the 1st examination	I	II	III	IV	V	VI	VII
Time rowed, min/day	-	-	150	90	80	100	40
Distance rowed, km/day	-	-	30	20	18	20	-
Training for force development, min/day	-	-	70	-	80	-	-
Low endurance rowing training time, min/day	80	-	130	65	80	100	40
High intensity endurance rowing training time, min/day	26	-	20	45	-	-	-
Unspecific training (running, etc.), min/day	30	80	20	10	30	10	10
Total training time, min/day	136	80	240	120	190	110	50
**Test II**							
Days before the 2nd examination	I	II	III	IV	V	VI	VII
Time rowed, min/day	100	80	100	120	0	90	70
Distance rowed, km/day	20	16	16	18	0	18	14
Training for force development, min/day	-	-	-	-	-	-	-
Low endurance rowing training time, min/day	88	80	84	90	0	80	70
High intensity endurance rowing training time, min/day	8	-	10	18	-	10	-
Very high intensity endurance rowing training time, min/day	4	-	6	12	-	-	-
Unspecific training (running, etc.), min/day	70	10	20	30	60	10	10
Total training time, min/day	170	90	120	150	60	100	80

**Table 2:** Anthropometric characteristics of study participants (N  =  15).

Variable	Supplemented (Mean ± SD)	Placebo (Mean ± SD)
Height [cm]	189.5 ± 3.6	190.0 ± 3.9
Body mass [kg]	85.8 ± 7.3	88.3 ± 7.0
Age [years]	20.1 ± 1.1	19.8 ± 0.7
Fat [%]	9.2 ± 2.4	11.1 ± 2.5
Lean body mass [kg]	77.9 ± 6.8	78.3 ± 5.0
Water [%]	64.8 ± 2.4	63.2 ± 1.5

**Table 3:** The dietary intake of food groups was assessed with 24-h recall and before the exercise test.

Parameter	Aronia Before	Aronia After	Placebo Before	Placebo After	P/P	A/A	P/A	P/AII
Food intake 24 h before tests								
Energy (kcal)	4134.29 ± 761.06	4249.57 ± 1329.12	4417.14 ± 852.40	4499.80 ± 747.68	0.47	0.44	0.34	0.44
Protein (g)	222.39 ± 43.67	199.31 ± 41.95	171.03 ± 34.51	167.23 ± 24.11	0.03	0.03	0.68	0.35
Fat (g)	128.37 ± 40.69	122.37 ± 37.37	113.50 ± 19.31	86.82 ± 12.99	0.61	0.01	0.59	0.036
Carbohydrate (g)	532.26 ± 119.92	601.09 ± 242.81	625.68 ± 95.51	786.90 ± 179.73	0.25	0.12	0.57	0.12
Fiber (g)	25.05 ± 11.72	29.19 ± 13.64	33.22 ± 10.03	48.09 ± 23.24	0.25	0.048	0.61	0.25
Food intake before exercise on the test day								
Energy (kcal)	1094.00 ± 414.39	986.00 ± 233.36	946.43 ± 429.17	1137.67 ± 368.33	0.65	0.48	0.61	0.48
Protein (g)	37.64 ± 11.89	55.06 ± 14.15	40.89 ± 27.17	60.11 ± 32.00	0.8	0.68	0.052	0.34
Fat (g)	30.85 ± 16.65	32.21 ± 12.32	25.29 ± 15.32	33.65 ± 11.39	0.58	0.78	0.88	0.77
Carbohydrate (g)	138.17 ± 38.42	120.25 ± 74.40	143.23 ± 57.68	150.90 ± 58.95	0.63	0.84	0.88	0.61
Fiber (g)	7.55 ± 3.57	6.02 ± 4.39	10.44 ± 5.84	7.92 ± 5.32	0.17	0.59	0.55	0.5

Footnotes: Values are presented as mean ± SD. P/P—between-group comparison (Aronia vs. placebo) before supplementation; A/A—between-group comparison (Aronia vs. placebo) after supplementation; P/A—within-group comparison before vs. after supplementation in the Aronia group; P/AII—within-group comparison before vs. after supplementation in the placebo group. Within-group comparisons were performed using paired Student’s *t*-tests, whereas between-group comparisons were performed using independent-samples *t*-tests. Statistical significance was set at *p* < 0.05. Dietary fiber intake was calculated from food records and did not include fiber derived from the placebo capsules as its exact quantity was unavailable.

**Table 4:** Summary of two-way ANOVA results for intestinal barrier-related biomarkers and cortisol.

Biomarker	ANOVA Effect	*p*-Value	ηp^2^
CLDN-3	Group × Time	0.0463	0.100
	Time	0.0251	0.203
	Group	0.74	0.009
LBP	Group × Time	0.0129	0.196
	Time	<0.0001	0.331
	Group	0.28	0.089
I-FABP	Group × Time	>0.05	0.042
	Time	<0.0001	0.527
	Group	0.18	0.13
Cortisol	Group × Time	0.0066	0.110
	Time	<0.0001	0.260
	Group	0.49	0.037

## Data Availability

The data supporting the findings of this study are available in the public repository at https://doi.org/10.18150/CKSZNQ. Additional data are available from the corresponding author upon reasonable request.

## Abbreviations

The following abbreviations are used in this manuscript:

CLDN-3	claudin-3
ELISA	enzyme-linked immunosorbent assay
GI	gastrointestinal
HPA	hypothalamic–pituitary–adrenal
I-FABP	intestinal fatty acid-binding protein
LBP	lipopolysaccharide-binding protein
RCT	randomized controlled trial
ROS	reactive oxygen species
